# Degradation and Mineralization of Phenol Compounds with Goethite Catalyst and Mineralization Prediction Using Artificial Intelligence

**DOI:** 10.1371/journal.pone.0119933

**Published:** 2015-04-07

**Authors:** Farhana Tisa, Meysam Davoody, Abdul Aziz Abdul Raman, Wan Mohd Ashri Wan Daud

**Affiliations:** Department of Chemical Engineering, Faculty of Engineering, University of Malaya,50603 Kuala Lumpur, Malaysia; Universitat Rovira i Virgili, SPAIN

## Abstract

The efficiency of phenol degradation via Fenton reaction using mixture of heterogeneous goethite catalyst with homogeneous ferrous ion was analyzed as a function of three independent variables, initial concentration of phenol (60 to 100 mg /L), weight ratio of initial concentration of phenol to that of H_2_O_2_ (1: 6 to 1: 14) and, weight ratio of initial concentration of goethite catalyst to that of H_2_O_2_ (1: 0.3 to 1: 0.7). More than 90 % of phenol removal and more than 40% of TOC removal were achieved within 60 minutes of reaction. Two separate models were developed using artificial neural networks to predict degradation percentage by a combination of Fe^3+^ and Fe^2+^ catalyst. Five operational parameters were employed as inputs while phenol degradation and TOC removal were considered as outputs of the developed models. Satisfactory agreement was observed between testing data and the predicted values (R^2^_Phenol_ = 0.9214 and R^2^TOC= 0.9082).

## Introduction

This can be achieved by in-depth investigation and implementing new treatment technologies. Phenol, as a widely used organic contaminant, can be found in various industrial wastewaters (i.e. petrochemical, paper-making, oil-refining, resin manufacturing, coking, and iron-smelting). Biological process cannot remove aromatic compounds such as phenol and benzene in many industrial effluents. Therefore, there is a growing interest in alternative treatment methods for degradation of highly toxic industrial wastewaters [1]. Use of biological and chemical process to treat phenol in wastewater to standard level (< 2ppm) is difficult due to its high solubility and stability in water [2,3]. Currently, there is increasing focus on absolute oxidation of organic compounds to nontoxic compounds. As a substitution of conventional processes, advanced oxidation processes (AOPs) have been much investigated in an attempt to degrade toxic compound completely. AOPs use catalytic and chemical photochemical methods to produce strong oxidizing radicals in acidic aquatic media. Fenton oxidation has been proven to be an efficient and powerful treatment process for mineralization of phenol compounds among AOPs [4–7]. AOPs can be generalized into homogeneous and heterogeneous processes depending on the physical state of the catalyst [2]. Hydrogen peroxide is the most widely used oxidant in AOPs. As hydrogen peroxide alone is not powerful enough in degradation of most organic compounds, normally a combination of hydrogen peroxide and iron salts or ozone is used for higher production of hydroxyl radical. The use of ferric ions with hydrogen peroxide is referred to as Fenton-like reaction [8].

Combination of goethite and hydrogen peroxide as an oxidant has been found to be satisfactory in oxidizing organic compounds for catalysis of goethite surface and ferrous ion generation. This Fenton-like process can be potentially used in treatment of toxic wastes as goethite is easily available in soil and can be recycled [4,8,9]. Based on the study of Ming Chun Lu, it was discovered that phenol degradation with goethite was slower and thus Fe^2+^ catalyst was used to accelerate the process [10]. Such organic contaminant treatment is complex because the process encompasses many numerical reactions influenced by a number of factors.

Modeling of these multivariate processes is comparatively complex [11–13]. Therefore, simple linear multivariate correlations are unable to solve these problems [14]. Previously presented works by some authors [11,13] on empirical mathematical modeling techniques have provided reference on well-performing mathematical models that can predict industrial process performance. The current knowledge base on waste water treatment is still limited, and therefore, hybrid AI architecture for the diagnosis, prediction and control of a wastewater treatment process could be employed to improve the operation of the treatment process.

Based on wide acceptance of ANNs application in engineering; artificial neural networks (ANNs) were used in this work as a predictive model [15–17]. The advantages of ANN are i) the mathematical description of the phenomena concerned in the process is not obligatory; ii) less time is necessary for model development and iii) prediction is possible with limited numbers of experiments [13,18]. There are several types of artificial neural networks and two of them are (i) multilayer feed-forward neural network trained by back propagation algorithm that is widely used, and (ii) Kohonen self-organizing mapping [19]. The feed forward is the most widely used method to map input-output relationship [20,21].

A number of research and review articles have been done on application of Artificial Neural Networks (ANN) analysis in environmental engineering problems. Some recent examples include modeling of dye removal by photo-Fenton process [11,14,20,22,23], industrial wastewater [24–26], pharmaceutical compounds [13] and fuel additives [27] treatments. ANN have also been notably appreciated for kinetic modeling [16] and automatic control system [28].

ANN Modeling for phenol degradation by Fenton process is new and there are limited relevant studies. Therefore, this work aims to assess the prospect of ANN Modeling in predicting catalytic activity of goethite catalyst in phenol and TOC removal. Two artificial neural networks were developed and optimized in order to predict phenol and TOC. The procedure allows us to consider parameter interdependencies and process unpredictability by encoding relationships between input and output variables [15]. In this study, relationships between the experimental variables (goethite catalyst, ferrous sulphate, hydrogen peroxide) and output variables, (TOC and phenol removal efficiency) were built through ANN. The last part of the study was devoted to indicating the relative importance of each proportional parameter on ANN.

## Material and Methods

### Experimental

Phenol 95% had been provided by Ranks and Synergy Sdn, 35% technical-grade H_2_O_2_, FeSO_4_.7H_2_O and H_2_SO_4_ 98% had been supplied by Merck. The goethite particles (α-FeOOH), anhydrous NaOH, anhydrous sodium sulphite Na_2_SO_3_, sodium phosphate NaH_2_PO_4_ and Potassium iodide (KI) employed in this work had been provided by Sigma Aldrich. The work was conducted in batch in a conical flux of 250 ml with good stirring at an rpm of 300. Phenol was added to distilled water to reach initial concentrations of 100 mg /L, 80 mg /L and 60 mg /L. After attaining steady-state temperature (25°C), the goethite catalyst was added in a weight ratio of 1: 20 to phenol concentration. After that, prescribed H_2_O_2_ and the required concentration of ferrous salt were added to the solution. The pH was adjusted with H_2_SO_4_ and NaOH and was monitored with Eutech pH 300 meter with pH electrode and ATC probe. The range of the initial amount of goethite catalyst loading and ferrous salt varied in three levels from 1.2 to 2 g/L and from 13.2 mg/L to 22.2 mg/L respectively. Each experiment lasted for 60 min. Nine different cases were investigated in destructing phenol in solution. Table 1 shows the list of experimental conditions employed.

**Table 1:** Applied run conditions for phenol degradation.

	Initial Phenol Conc. (mg/L)	Initial FeSO_4_.7H_2_O Conc. (mg/L)	Initial Goethite Conc. (g/L)	Initial H_2_O_2_ Conc. (mg/L)
Case 1	100	22	2	600
Case 2	80	17.6	1.6	480
Case 3	60	13.2	1.2	360
Case 4	100	22	2	1000
Case 5	80	17.6	1.6	800
Case 6	60	13.2	1.2	600
Case 7	100	22	2	1400
Case 8	80	17.6	1.6	1120
Case 9	60	13.2	1.2	840

15 ml of solution was collected from the reactor at certain time intervals in the experiments. The samples were treated with equal volume of a reaction stopping reagent (0.1 mol/L Na_2_SO_3_, 0.1 mol/L NaH_2_PO_4_, 0.1 mol/L KI and 0.5 mol/L NaOH) to terminate the oxidation process and prepare them for analysis. Total Organic Carbon (TOC) and phenol concentration analysis were done for each sample. Phenol concentration was monitored with HPLC (Shimadzu) equipped with a LC-18 C18 column. The mobile phase was an isocratic mixture of water (containing 0.4 mM of sulphuric acid), pumped at a rate of 1 mL/min with retention times of 30 min for phenol. Phenol was detected at wave length of 225 nm. TOC was measured with an Aurora TOC-Analyzer. Solid particles were filtered with whatman syringe filters of 0.02 μm for HPLC and TOC analysis. Phenol removal in Fentonic AOP treatment can be calculated as:

$$\text{Phenol removal efficiency }=\frac{{\left[\text{Phenol conc}.\right]}_{\text{o}}-{\left[\text{Phenol conc}.\right]}_{\text{t}}}{{\left[\text{Phenol conc}.\right]}_{\text{o}}}$$ (1)

Where, [Phenol conc.]_o_ is the initial concentration and [Phenol conc.]_t_ is concentration at time t. The TOC removal can be calculated as follows,

$$\text{TOC removal efficiency }=\frac{{\left[\text{TOC}\right]}_{\text{o}}-{\left[\text{TOC}\right]}_{\text{t}}}{{\left[\text{TOC}\right]}_{\text{o}}}$$ (2)

Where, [TOC]_o_ is the initial concentration and [TOC]_t_ is concentration at time t.

### Artificial Neural Networks Software

Artificial Neural Networks (ANN) is a computational system which follows the computational abilities of biological systems. ANN creates a network consisting of multiple layers with artificial neurons to determine the relationship between the experimental data. These neurons are simple processing elements which transfer the received data to output through the simple equation below [29]

$$O_{i}=f\sum_{j=1}^{n}w_{i}{}_{j}I_{j}+b_{i}$$ (3)

where *O*_*i*_, *f*, *w*_*ij*_, *I*_*j*_, *b*_*i*_, and *n* refer to the output of the *i* ^th^ neuron, transfer function, synaptic weight corresponding to *j* ^th^ synapse of *i* ^th^ neuron, *j* ^th^ input signal to the *i* ^th^ neuron, bias of the *i* ^th^ neuron, and the number of input signals to the *i* ^th^ neuron, respectively. The neurons inside the network are connected to each other by a direct communication link with associated weight (*w*_*ij*_).

In this study, two separate ANN models were developed to map the effective inputs to targets. The considered input variables were H_2_O_2_, Fe^2+^ initial concentration, phenol initial concentration and catalyst. The outputs of the models were time of phenol conversion and TOC. For simplicity, the terms-phenol model and TOC model, were used throughout this report to refer to each model. It has been proposed previously that having one or more hidden layers enables the network to model most non-linear data behaviors [30,31]. Therefore, two multilayer feed-forward ANNs with one hidden layer were developed for approximation. In both networks, sigmoid and linear transfer functions were considered for the hidden and output layers, respectively. Both networks were trained using Levenberg-Marquardt back-propagation algorithm. All ANN calculations were carried out using Matlab 6.5 with ANN toolbox for windows which were run on a personal computer (Pentium IV 2800 MHz). The available data was randomly split into three categories—train, test, and validation groups.

## Results and Discussion

### Phenol degradation with Fe^3+^ and Fe^2+^ catalyst

It has been denoted that use of goethite catalyst can be effective in diminution of organic complexes [8,9,32]. Goethite and hydrogen peroxide can effectively oxidize organic compounds. Hence, batch experiments were performed in conical bottles with 100 mg/L phenol and 2 g/L goethite at initial pH 3 to examine the reductive oxidation effect of phenol by goethite. No degradation was observed within 60 min by using goethite alone; but highly efficient degradation was achieved with the use of ferrous sulphate. In a study of Ming-Chun Lu (1999), degradation of chlorophenol using only goethite catalyst was observed within 3 to 4 hours [8]. When goethite catalyst is used in Fenton oxidation, ferrous ions are produced from the reductive dissolution of goethite as shown below [8,33]

$$\alpha -FeOOH+3H^{+}+e^{-}↔Fe^{2+}+3H_{2}O$$ (4)

Electron is generated from hydrogen peroxide from the equation below,

$$H_{2}O_{2}\to 2H^{+}+O_{2}+2e^{-}$$ (5)

Reaction 4 and 5 can be simplified to reaction 6,

$$\alpha -FeOOH+2H^{+}+\frac{1}{2}H_{2}O_{2}\to Fe^{2+}+\frac{1}{2}O_{2}+2H_{2}O$$ (6)

The degradation would follow the classic steps corresponding to the Homogeneous Fenton reactions, among which are:

$$Fe^{2+}+H_{2}O_{2}\to Fe^{3+}+HO^{-}+HO^{o}$$ (7)

$$Fe^{3+}+H_{2}O_{2}\to Fe^{2+}+H^{+}+HO_{2}$$ (8)

**Fig 1:**
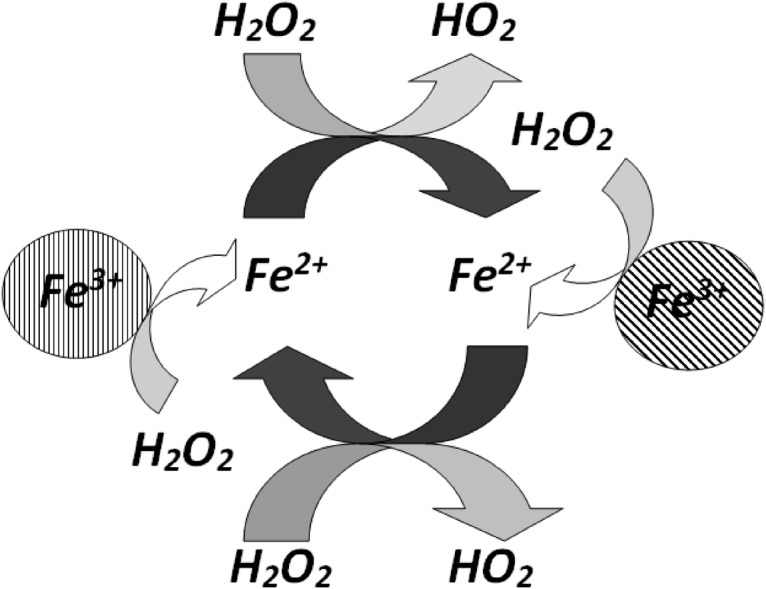
Mechanism Description.

The mechanism of phenol degradation can be explained by the above equations. Based on the literature, oxidation of phenol with H_2_O_2_ in presence of Fe^2+^ catalyst occurs when there is electrophilic attack by hydroxyl radical. A more complete mechanism can be found in work of authors [1,34]. A simplified mechanism is shown in Fig. 1.

The degradation of phenol compound is complex. Phenol oxidation has been studied by various authors as means of producing principal intermediates (e.g. benzoquinone and hydroquinone). However, there is little known about the sequence of intermediates formation. Thus, Total Organic Carbon (TOC) is taken as a surrogate parameter of organic matter present in the water and considered as a sum of contribution of the organic compounds.

**Fig 2:**
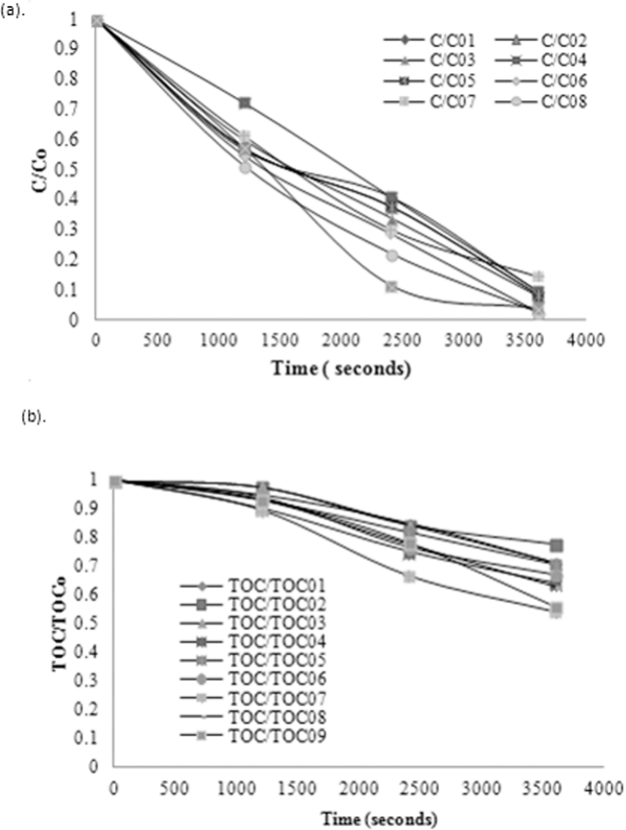
Degradation trend for all experimental conditions (a) Phenol conversion, (b) TOC removal.

Fig. 2 shows the experimental results in dimensionless concentrations vs. time for experimental runs 90% phenol conversion [phenol: H_2_O_2_ = 1: 6 or 1: 10 or 1: 14] was observed in the experiment. An increase in phenol degradation was attained with time on average. It is quite notable that even though there was a significant decrease in phenol concentration in the solution with time, the same degradation rate was not observed for TOC of the solution. This incidence can be related to noticeable increase in intermediate products (maleic acid, benzoquinone, hydroquinone etc) [35]. Choi J-S, Yoon S-S, Jang S-H, Ahn W-S, investigated the initiation phase for phenol hydroxylation using Fe containing catalysts and the result depicted that it occurred during the first 5–15 min [36], which was also the time required for reaction (6) to (8) to take place. Hydroxylation progressed via a redox mechanism involving Fe (III)/ Fe (II) redox pair [36].

**Fig 3:**
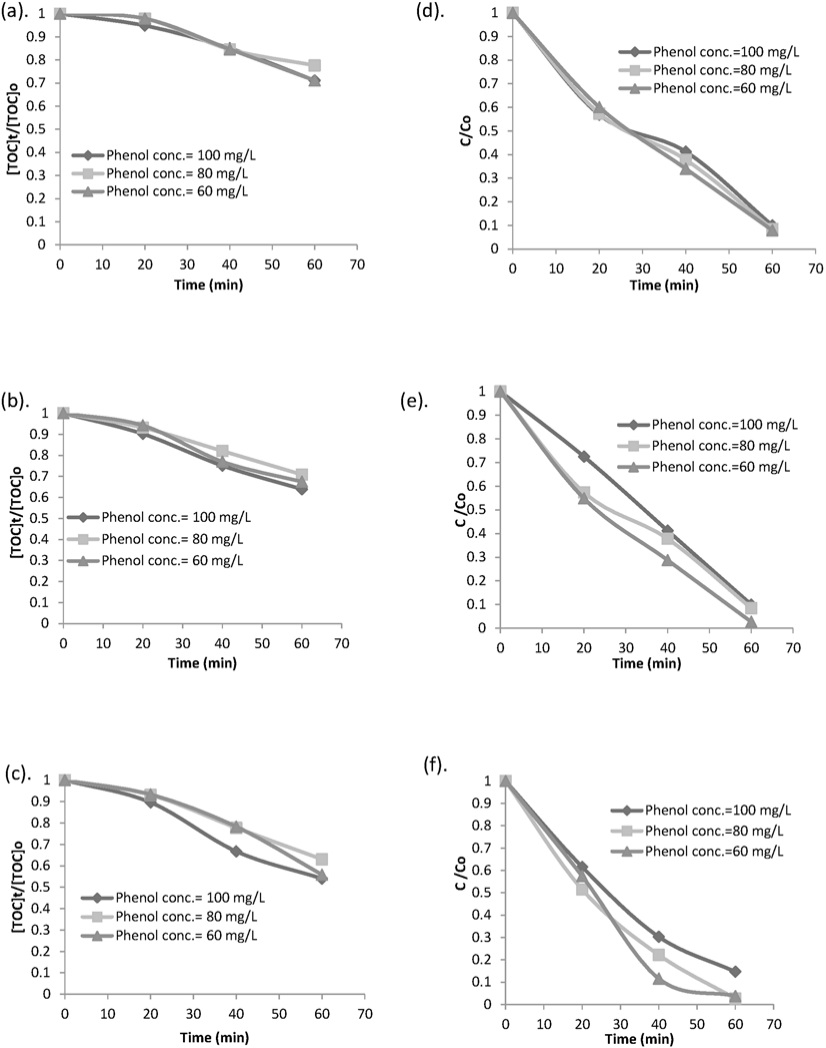
Effect of initial phenol concentration on TOC removal and phenol oxidation at different Phenol conc.: Hydrogen peroxide conc. ratios (a),(d) [Phenol]_0_: [H_2_O_2_] = 1: 6, (b),(e) [Phenol]_0_: [H_2_O_2_] = 1: 10 and (c), (f) [Phenol]_0_: [H_2_O_2_] = 1: 14.

A number of competing factors may possibly help acquire the best possible degradation and mineralization rate. Firstly, hydroxyl radicals produced for phenol mineralization are available with enhancement of total iron availability in the solution. The mechanism follows (equation 7) and (8). Secondly, the reaction between ferric ions and hydrogen peroxide that produces HO_2_ radical, which is comparatively much less reactive than hydroxyl radical, becomes vital at a higher total iron concentration [37]. Besides, the scavenging reactions which become predominant in presence of high amount of hydrogen peroxide and catalyst, affect the degradation and mineralization rate. In this study, phenol mineralization was not obtained in absence of iron which can be supported by the fact that no degradation takes place when there is only hydrogen peroxide. The degradation and mineralization rate could be observed over time in Fig. 3. Based on this figure, higher mineralization and degradation were observed for comparatively lower initial phenol concentration at the same ratio of phenol to hydrogen peroxide concentration.

### Effect of initial hydrogen peroxide concentration

**Fig 4:**
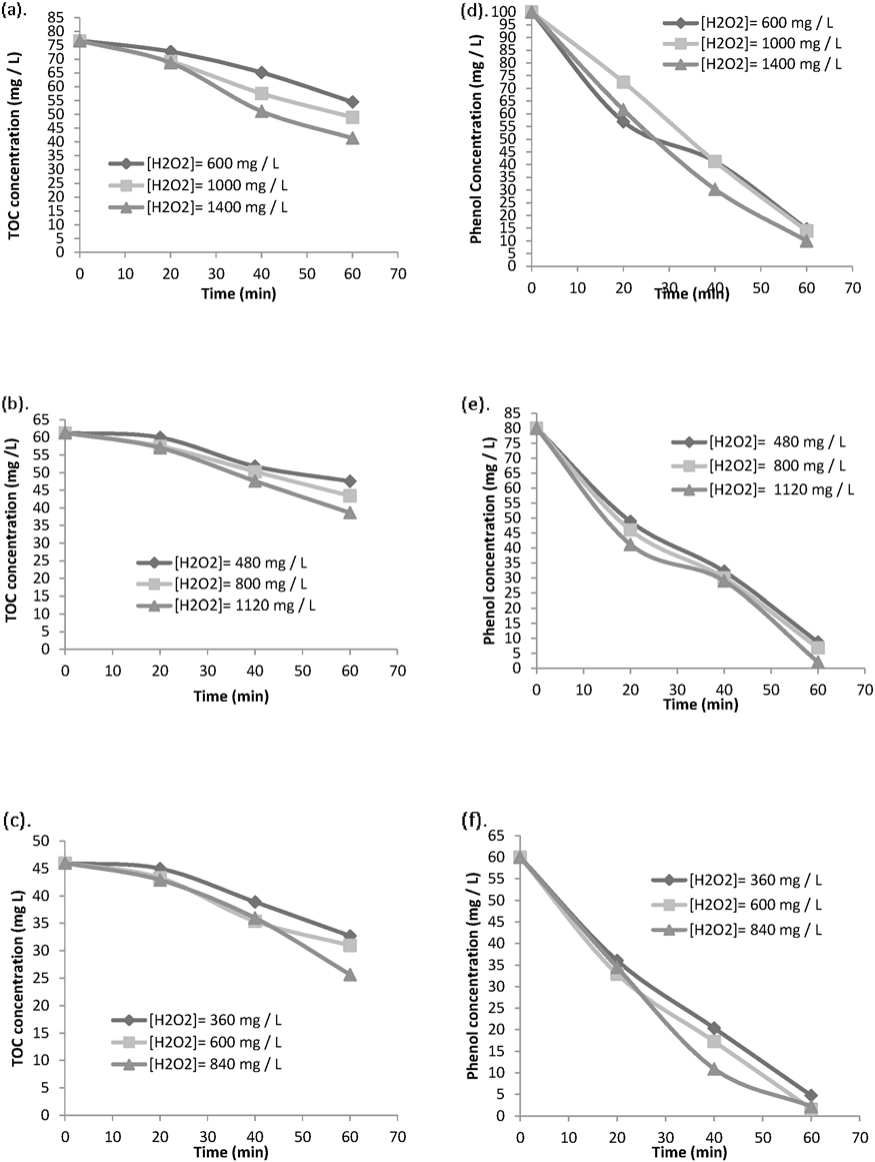
Effect of initial hydrogen peroxide concentration on TOC removal and phenol oxidation at different Phenol conc.(a),(d) [Phenol]_0_ = 100 mg / L(b),(e) [Phenol]_0_ = 80 mg / L and (c), (f) [Phenol]_0_ = 60 mg / L.

Concentration of hydrogen peroxide is an important parameter for phenol degradation. Thus, influence of amount of hydrogen peroxide was investigated. Different ratios of phenol: hydrogen peroxide was investigated for 100mg/L of phenol destruction. The ratio differed from 1: 6 to 1: 14. Based on the result, it can be concluded that ratio of phenol: hydrogen peroxide of 1: 14 produced the best degradation and mineralization rate. Usually, the amount of H_2_O_2_ used is greater than the stoichometry amount necessary for complete mineralization of the initial organic compound [38]. One requires at least 506 mg /L of H_2_O_2_ in order to completely mineralize 100 mg /L of phenol, but 85% mineralization was achieved by Huang C-P and Huang, Yao-Hui with ratio of phenol to hydrogen peroxide of 1: 40 in mg /L [39]. Higher removal of TOC is probably due to direct oxidation of phenol or its intermediates. Generally, direct oxidation process of phenolic compounds to desired end products (carbon dioxide and water) is very slow. Phenoxy radicals, polymer species, benzoquinone, muconic, maleic, formic, oxalic acids have been reported as the intermediates formed in mineralization process of phenol. In our research with application of goethite catalyst along with ferrous solution, the ratio of hydrogen peroxide to initial phenol concentration was decreased to 1: 14 with > 90% phenol removal in 60 min. No mineralization was observed within 150 min with goethite catalyst alone. In the work of Zazo JA, Casas JA, Mohedano AF and Gilarranz MA, Rodiguez JJ ratio of ferrous to hydrogen peroxide of 1: 500 mg /L resulted in less than 50% mineralization of phenol with initial TOC of 76.6 mg /L [34]. > 30% removal of TOC was achieved with addition of 2 g /L goethite catalyst and ferrous salt in a weight ratio of 1: 0.22 per 100 mg /L of phenol in our research in less than 100 min. It can be depicted from the observed result that, the kinetic rate and mineralization greatly depend on initial concentration of hydrogen peroxide. The degradation rate of hydrogen peroxide maintains a linear correlation with mineralization of phenol. Fig. 4 represents the degradation and mineralization trend for various ratios of initial phenol concentration to hydrogen peroxide concentration.

### Effect of Goethite catalyst and oxidant ratio

**Fig 5:**
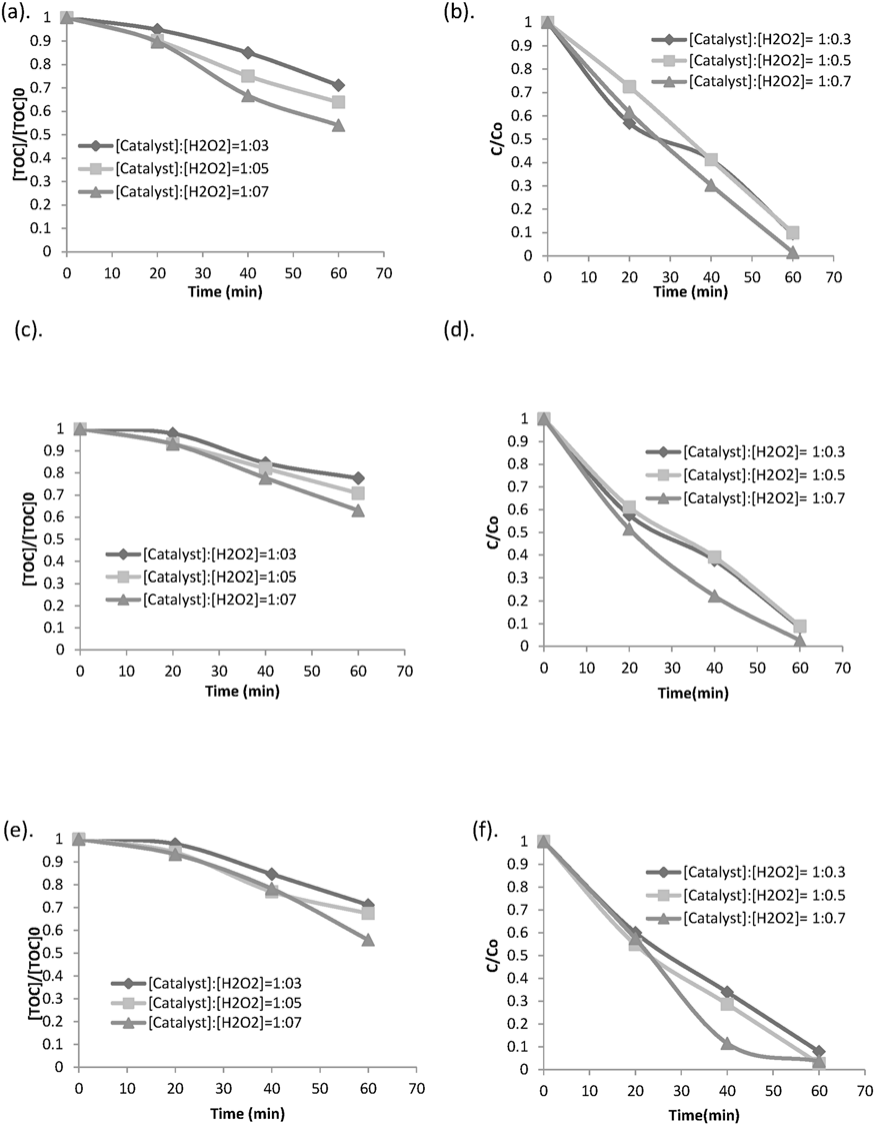
Effect of initial catalyst and Hydrogen peroxide ratios on TOC removal and phenol oxidation (a),(d) [Phenol]_0_ = 100 mg / L, (b),(e) [Phenol]_0_ = 80 mg / L and (c), (f) [Phenol]_0_ = 60 mg / L.

Phenol in concentration of 100 mg /L was degraded with various catalyst oxidant ratios to determine the effect of goethite catalyst. A constant ratio of ferrous ion to initial phenol concentration was maintained. However, different mineralization rates were observed with changes in goethite and oxidant ratio. It was observed that the degradation rate increased by increasing ratio from 1: 0.3 to 1: 0.7. However, surprisingly, the degradation rate decreased with more hydrogen peroxide. This can be explained from the fact that with increase in hydrogen peroxide to Fe^3+^ catalyst ratio, more hydrogen peroxide is consumed in production of Fe^2+^ from Fe^3+^ compared to from mineralization of phenol. It can also be assumed that scavenging effect is one of the causes for decreased degradation rate. [40,41]. In acidic condition, phenol mineralization is more expeditious in the existence of ferrous sulphate. Generally, decrease in solution pH usually leads to intermediates formation (i.e. carboxylic acid, oxalic acid and formic acid) [39]. Fig. 5 shows phenol degradation and mineralization rate for different catalyst and oxidant ratios.

### Neural network modeling

Learning algorithms adjust values of weight and bias while designing a neural network. Selecting appropriate learning algorithm and transfer functions is vital for a reliable neural network. Since there is not any reliable method to determine the optimum values, the best learning algorithms and the associated transfer functions were determined by trial and error. Therefore, a range of options were studied in order to choose the best algorithm and transfer functions. Levenberg-Marquardt and Scaled Conjugate Gradient were considered as learning algorithms while the training functions and the transfer functions of the layers varied according to the applied algorithm. It has been reported that 8–11 neurons in hidden layer would produce the minimum value of mean square error (MSE) [42]. Therefore, number of hidden layer neurons was maintained at 8 in the present study. Table 2 compares the efficiency of the built models in difference scenarios. Mean square error (MSE) was used to determine the performance of the developed networks. MSE was calculated based on the following equation:

$$MSE=\frac{1}{N}\sum_{i=1}^{N}{\left(T_{i}-O_{i}\right)}^{2}$$ (9)

**Table 2:** Results of trial and error method to determine optimum learning algorithm and transfer functions.

Model	Back-propagation algorithm	Training function	Transfer function		R^2^ (test)	MSE×10^3^ (test)
			Hidden layer	Output layer		
Phenol	Levenberg-Marquardt	**trainlm**	satlin	purelin	0.3994	60.91
				tansig	0.8933	9.4011
			poslin	purelin	0.8419	11.033
				tansig	0.9119	8.892
			**tansig**	**purelin**	**0.9214**	**7.661**
				tansig	0.7328	20.287
	Scaled Conjugate Gradient	trainscg	purelin	purelin	0.6891	24.438
				tansig	0.8973	10.09
			poslin	purelin	0.7633	18.79
				tansig	0.3966	59.38
			tansig	purelin	0.9154	7.89
				tansig	0.7155	23.81
TOC	Levenberg-Marquardt	**trainlm**	satlin	purelin	0.8246	14.578
				tansig	0.6957	23.743
			poslin	purelin	0.8707	12.02
				tansig	0.7521	22.39
			**tansig**	**purelin**	**0.9082**	**9.026**
				tansig	0.8139	17.123
	Scaled Conjugate Gradient	trainscg	purelin	purelin	0.7712	16.19
				tansig	0.4206	54.18
			poslin	purelin	0.6891	24.438
				tansig	0.8685	12.977
			tansig	purelin	0.6291	29.78
				tansig	0.7678	19.21

Each run was repeated three times to minimize the chance of random correlation due to random initialization of the weights. Based on the values of the last two columns, Levenberg-Marquardt algorithm with tansig and purelin transfer functions generated the most accurate network for both models (R^2^_Phenol_ = 0.9214, R^2^_TOC_ = 0.9082). Therefore, this structure was considered for further optimization. As for Phenol model, changing the learning algorithm from Levenberg-Marquardt to Scaled Conjugate Gradient did affect the accuracy of predictions significantly as long as the transfer functions of both layers were constant (R^2^_Phenol_ = 0.9154). Similar update onto the network of TOC model produced a huge impact on the performance of the model (R^2^_TOC_ = 0.6291). Regardless of the transfer functions used, TOC model showed relatively poor performance when the weights of the network were adjusted by Scaled Conjugate Gradient. It was concluded that TOC model was much more sensitive to change in learning algorithm compared to Phenol model.

After identifying the algorithm and transfer functions, attention was focused on improving the performance of models through updating the number of neurons in hidden layers. Number of layers and number of transfer functions in each layer are the key parameters in determining the topology of a neural network. Optimization of the generated topology is a major challenge in building ANN models. High number of neurons in the hidden layer (hidden neurons) may cause the network to learn from the training data but fail to perform properly in the validation part, a problem known as ‘overfitting’, while employing fewer neurons could waste a considerable training time in finding the optimal representation [43]. Different number of hidden neurons creates different networks with different performances, and the optimum number is selected based on comparison between the prediction errors of the created networks. In this study, different topologies were created by varying the number of hidden neurons from 2 to 20. Each topology was run three times and the average values were used to determine the accuracy of the results. Error of networks is calculated by the (Equation 9).

Where N, T, O, and i refer to number of data sets, targets (experimental values), outputs (model’s predictions), and index of data, respectively.

**Fig 6:**
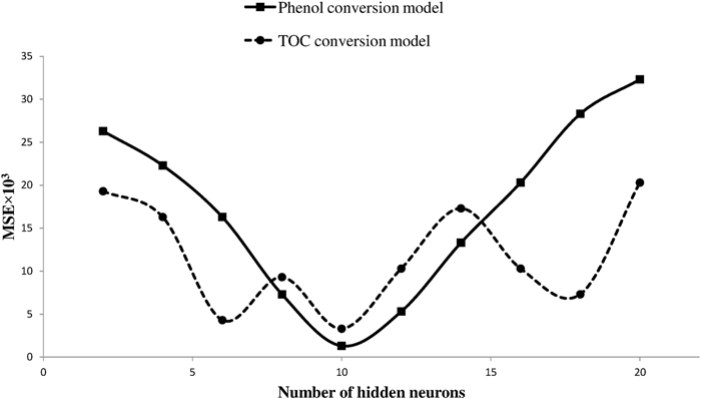
Effect of number of hidden neurons on network performance.

**Fig 7:**
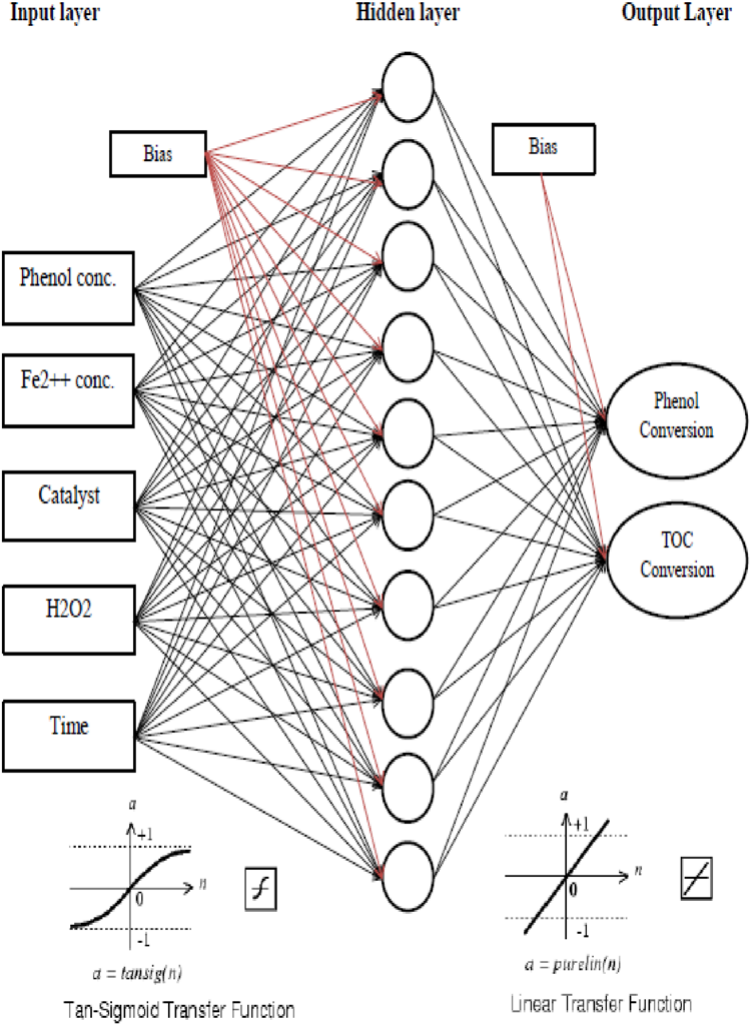
Optimum neural networks for Phenol and TOC models.

The network errors are plotted versus number of neurons in hidden layer for Phenol (straight line) and TOC (dotted line) models in Fig. 6. As shown in Fig. 6, both models had the least mean square error values when 10 nodes were used in their hidden layers. Fig. 7 depicts the structure of a three-layer feed-forward back-propagation neural network consisting of ten hidden neurons. The structure was almost the same for both models with the output variable being the only difference. Fig. 7 is followed by Table 3 which lists the specifications of the proposed models.

**Table 3:** Specifications of the proposed models.

Parameter	Number/Type	Details		
		Variable	Maximum	Minimum
Input	5	Phenol conc. (mg / L)	100	60
		Fe^2+^ conc. (mg / L)	22	13.2
		Catalyst (g/L)	2	0.6
		H_2_O_2_ conc. (mg / L)	1400	360
		Time (min)	60	1
Output	2	Phenol conversion (%)	102.621	-2.175
		TOC conversion (%)	49.681	-5.9587
Architecture	Feed-forward	Connections between the units do not form a directed cycle [44]		
Training algorithm	Levenberg-Marquardt backpropagation	Updates weight and bias values according to Levenberg-Marquardt optimization [45]		
Database		Division (randomly)		
		Train	Test	Validation
		70%	15%	15%
Epoch number	400			
Hidden layer	1	Number of neurons	10	

**Fig 8:**
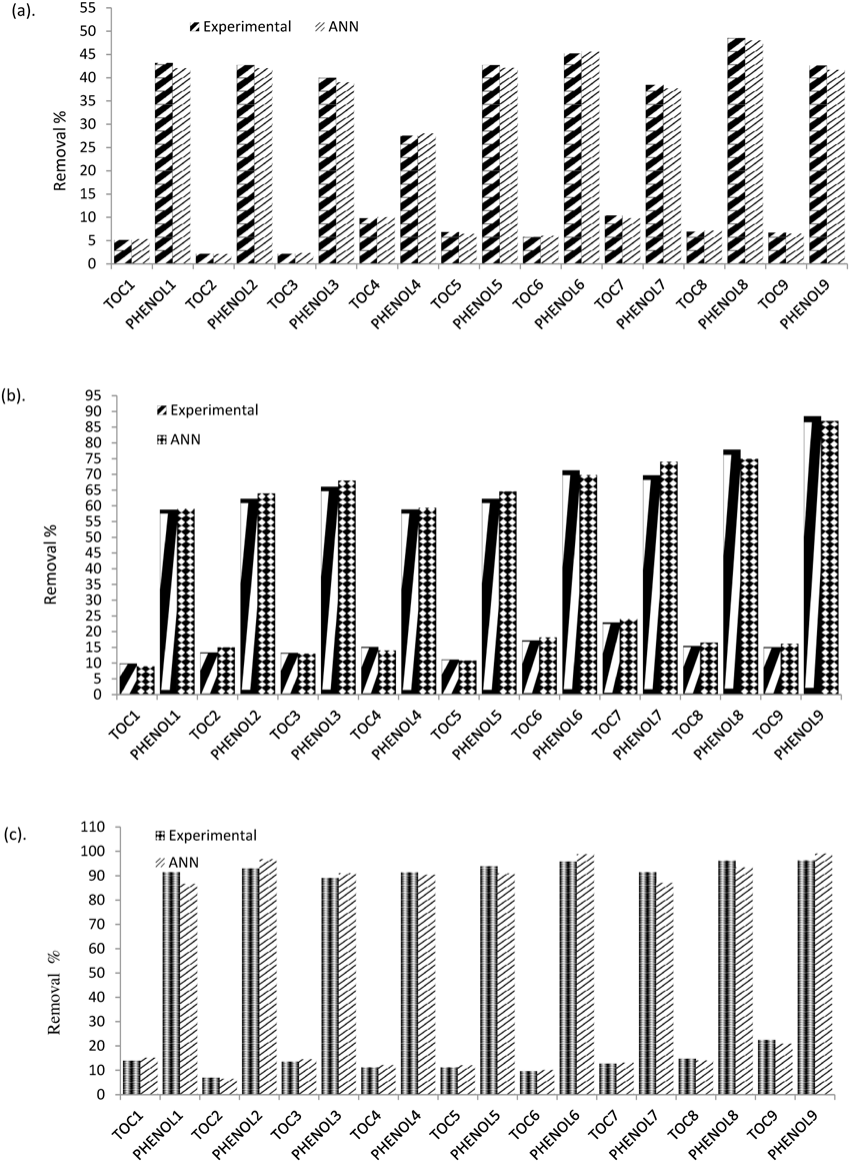
Comparison between experimental and neural networking results for TOC removal % and Phenol degradation % (a.) 20 min time interval, (b.) 40 min time interval and (c.) 60 min time interval.

Performance of the proposed models was evaluated by comparing their predicted values with the experimental values. Data of the test group was used for this purpose. Correlation coefficients of R^2^ = 0.976 and R^2^ = 0.968 were achieved for Phenol and TOC models, respectively, which showed acceptable agreement between the outputs and the corresponding real values of both model. Fig. 8 presents a graphical comparison between the experimental data obtained after 20 minutes and the corresponding ANN predictions.

### Sensitivity analysis

In this part of study sensitivity analysis (SA) was carried out to investigate the degree of importance of input parameters on the model outputs. The results of this analysis can provide useful information on the sensitivity of input variables and their robustness, and therefore, can lead to a better decision making process.

Weight matrix and PaD method are widely applied on ANN models to analysis the sensitivity of input parameters [46]. Both methods are used in this study to determine the degree of influence of each input variable on outputs.

#### Weight Matrix

By having ten nodes in the hidden layer, a total of 71 weights have been calculated, out of which 60 weights are between input and hidden layers (5 weights associated with 5 input variables plus one bias for each of the hidden neurons) and remaining 11 weights are between hidden and output layers (10 weights associated with 10 nodes plus one bias). The corresponding weights are listed in Table 4 (matrix of weights between input and hidden layers) and Table 5 (matrix of weights between hidden and output layers).

**Table 4:** Matrix of weights between input and hidden layers.

Neuron	Model	N_1_	N_2_	N_3_	N_4_	N_5_	N_6_	N_7_	N_8_	N_9_	N_10_
Weights associated with Phenol	Phenol	-0.40796	1.2906	-1.2523	1.6452	-2.7691	1.4834	-0.34388	-3.1652	-1.6316	-2.9351
	TOC	0.49664	-3.4066	-1.8139	2.1911	4.2199	0.8811	2.3809	-1.7433	1.996	-3.2118
Weights associated with H_2_O_2_	Phenol	4.3705	2.1813	-0.86459	2.6222	1.5193	-2.8122	2.2421	0.47327	-2.3254	-3.2886
	TOC	4.8311	-3.8146	-2.6098	3.45661	-1.9155	3.25782	-2.6148	2.0078	0.96543	1.3433
Weights associated with Catalyst	Phenol	-0.80785	-3.7103	2.1372	-3.2521	4.7338	-1.1942	2.8819	2.1722	-2.306	-3.6056
	TOC	-0.84554	-1.73443	1.6905	1.7555	3.1096	-1.9576	0.8511	-3.5744	2.0447	-3.2319
Weights associated with Fe^2^	Phenol	-0.81306	-3.5148	-2.5857	-2.3766	2.1828	5.6628	-6.3257	0.78139	-4.8593	-2.7868
	TOC	0.3287	1.1877	-3.0211	-1.5307	-2.7966	-1.37557	-0.29791	-1.1433	1.49887	2.6487
Weights associated with Time	Phenol	-0.80989	-3.6098	-2.1892	-2.7754	3.3289	4.08733	-5.8867	1.4577	-3.7122	-3.011
	TOC	2.0382	-1.5806	2.7365	-1.4677	-2.9001	0.78076	1.4311	-2.4719	4.1055	2.4249
Bias	Phenol	1.9282	-1.3316	2.5132	-1.2361	-2.8601	0.56584	1.2038	-2.2529	3.9863	2.249
	TOC	-2.1066	3.191	-0.59987	1.6766	1.2266	1.81887	3.8832	2.2366	-2.5796	1.4298

**Table 5:** Matrix of weights between hidden and output layers.

Neuron	N_1_	N_2_	N_3_	N_4_	N_5_	N_6_	N_7_	N_8_	N_9_	N_10_
Weight (Phenol)	-0.82118	0.10765	0.1383	0.44851	-0.04487	0.27667	-0.07889	0.10876	0.1533	0.67654
Bias	1.0029									
Weight (TOC)	-0.73409	-0.09307	-0.1198	-0.39833	-0.1055	0.07012	0.05876	0.0081	0.1055	0.39699
Bias	1.0033									

**Fig 9:**
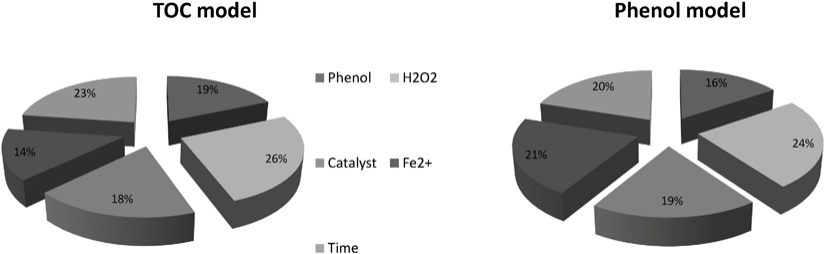
Relative importance of input variables on (a) Phenol conversion model, (b) TOC removal model.

Studies on weight matrix are necessary for evaluating the relative importance of each input variable on output variable. In this regard, the following equation which is based on the partitioning of connecting weights was mainly used [47] in this study:

$$I_{g}=\frac{\sum_{m=1}^{m=N_{h}}\left(\left(\frac{\left|W_{gm}^{ih}\right|}{\sum_{k=1}^{N_{i}}\left|W_{km}^{ih}\right|}\right)\times \left|W_{mn}^{ho}\right|\right)}{\sum_{m=1}^{k=N_{i}}\left\{\sum_{m=1}^{m=N_{h}}\left(\left(\frac{\left|W_{gm}^{ih}\right|}{\sum_{k=1}^{N_{i}}\left|W_{km}^{ih}\right|}\right)\times \left|W_{mn}^{ho}\right|\right)\right\}}$$ (10)

where I_g_, N_i_, N_h_, W, i, h, o, k, m, and n, refer to the relative impact of the g-th input variable on the output variable, number of input neurons, number of hidden neurons, connection weight, input layer, hidden layer, output layer, input neuron number, hidden neuron number, and output neuron number, respectively. Fig. 9 represents the relative importance of the input variables for both models.

According to Fig. 9, H_2_O_2_ had the highest impact on outputs of both models. Moreover, the calculated values listed in Fig. 9 show that all five input variables had considerable impacts on the output of both models. Hence, none of the investigated parameters could be ignored in the present modeling. The ANN models delivered a strong means of prediction with slight error and the predictions was smooth, over the range of data sizes used in training and testing.

#### PaD method

In this part PaD method was considered for conducting SA on the finalized ANN model. Satisfactory performance of this technique has been reported in previous studies [46,48–50].

In this method sensitivity of the input variable is determined by the following equation [46]:

$$S_{i}=\frac{1}{N}\sum_{P}\frac{\partial O_{{}_{k}}^{p}}{\partial X_{i}^{P}}$$ (11)

Here, n, p, $O_{k}^{p}$, $X_{i}^{p}$, correspond to the number of data variables, pattern number, the output value for the pattern p, and the input value from pattern p, respectively. o_k_ and o_j_ can be calculated by the following formula:

$$o_{K}=f_{2}\left(\sum_{j}w_{kj}o_{j}\right)$$ (12)

$$o_{j}=f_{1}\left(\sum_{i}w_{ij}x_{i}\right)$$ (13)

Where *w*_*kj*_, *w*_*ij*_, *o*_*j*_, *f*_*1*_ and *f*_*2*_ refer to the weight between output neuron *k* and the hidden neuron *j*, the weight between the input neuron *i* and hidden neuron *j*, the output of the hidden neuron *j*, and the activation functions, respectively.

Combination of (Equation 12) and (13) and incorporation of them to (Equation 11) results in (Equation 14):

$$S_{i}=\frac{1}{N}\underset{p}{\sum^{}}\frac{\partial o_{k}}{\partial o_{j}}\frac{\partial o_{j}}{\partial x_{i}}=\frac{1}{N}\underset{p}{\sum^{}}f_{2}^{℩}\left(\underset{j}{\sum^{}}w_{kj}o_{j}\right)\underset{j}{\sum^{}}w_{kj}f_{1}^{℩}\left(\underset{i}{\sum^{}}w_{ij}x_{i}\right)w_{ij}$$ (14)

Since the activation functions are sigmoid:

$$f^{1}=f\left(1-f\right)$$

And (Equation 14) can be updated to (Equation 15):

$$S_{i}=\frac{1}{N}\sum_{p}o_{k}\left(1-o_{k}\right)\sum_{j}w_{kj}o_{j}\left(1-o_{j}\right)w_{ij}$$ (15)

Moreover, the relative contribution of input parameters can be calculated by computing the sum of the squares of the partial derivatives:

$$SSD_{i}=\sum_{P}{\left(\frac{\partial o_{{}_{k}}^{p}}{\partial x_{i}^{P}}\right)}^{2}$$ (16)

And contribution of each input parameter is given by:

$$Contributionofithvariable=\frac{SSD_{i}}{\sum_{i}SSD_{i}}$$ (17)

**Fig 10:**
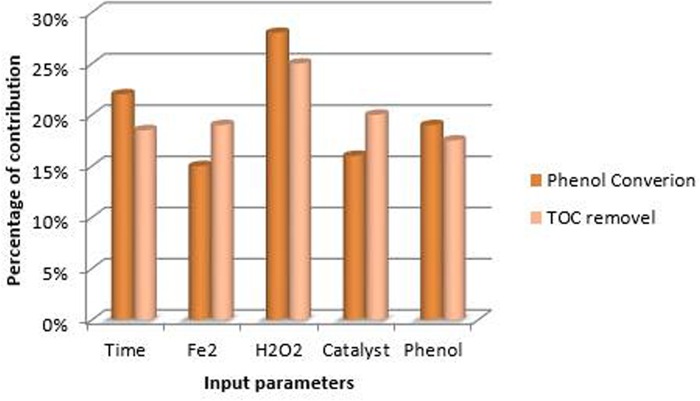
Contribution of input parameters on outputs based on PaD method.

Higher value of SSD indicates higher influence of the input variable on the output. Therefore, input variables can be ranked according to their impact on the target. Fig. 10 presents the contributions of the input variables based on PaD sensitivity analysis.

As it can be seen in Fig. 10, H_2_O_2_ is the most significant parameter on both targets while Phenol is the least. The contribution values reported by PaD method are slightly different compared to the ones obtained by weight matrix. Yet, both methods identify same critical parameters.

### Comparison with the previous models

In this section performance of the proposed ANN model is compared with the previous neural networks developed in other studies. Table 6 compares the ANN models in literature with the one designed in this study in terms of input/output parameters, magnitude of data set, and prediction accuracy.

**Table 6:** Comparison between the available ANN models in literature and the finalized neural network in this work.

Ref	Input parameters	Output parameter(s)	Experimental sets fed to ANN	Performance
[11]	[Dye]_0_	photochemical decolorization	228	MSE = 4*10^-3^
	[H_2_O_2_]_0_			R^2^ = 0.996
	pH			
	Time			
[14]	[H_2_O_2_]_0_ / [Dye]_0_	Degradation efficiency	100	MSE = 3*10^-2^
	Catalyst			R^2^ = 0.996
	pH			
	[Dye]_0_			
[20]	pH	Decolorization efficiency	232	MSE = 1.56*10^-4^
	Time			R^2^ = 0.9984
	[H_2_O_2_]			
	[Fe(II)]			
	[DR16]_0_			
	Temperature			
[22]	[H_2_O_2_]	Mineralization rate pseudoconstant	46	Average error lower than 11%
	[Fe]			
	Air flow rate			
	[COOH]_2_			
	pH			
[23]	[H_2_O_2_]	Decoloration constant	46	Average error lower than 18%
	[Fe(II)]			
	[RB4]	mineralization constant		Average error lower than 14%
	pH			
	Temperature			
This study	Phenol	Phenol conversion	30	MSE = 2*10^-3^
	[H_2_O_2_]			R^2^ = 0.976
	Catalyst	TOC removal		MSE = 4*10^-3^
	Fe^2+^			R^2^ = 0.968
	Time			

While general performance of the finalized neural network in this study is acceptable, it exhibits lower R^2^ values compared to [11] [14] [20]. The main reason for this difference can be the limited number of experimental data that was used to train networks in the current work. High number of experimental sets (considered for training ANN) can positively influence the ability of neural networks to understand behavior of the system under study and update their learning parameters. As such, networks trained with 70% of 200 sets can provide more accurate predictions compared to the ones trained with 70% of 30 sets. Nonetheless, the finalized ANN model offers more accurate predictions compared to the models that were developed with almost similar number of training sets in references [22] and [23].

## Conclusions

The vital principle of this paper was to analyze effect of parameters in the process of phenol degradation with Fe^3+^ and Fe^2+^ catalysts and application of ANN technique to model the process. The conclusions of the work can be stated as below:

1. Phenol was successfully degraded by goethite with combination of Fe^2+^ catalyst and hydrogen peroxide. After 1 h of reaction, the maximum percentage of phenol degradation for the number of experiments was more than 90% with maximum mineralization rate of over 40%. The results verified that this heterogeneous Fenton reaction is an efficient process for the degradation of phenol in aqueous solution.
2. Simulations based on the ANN model were performed in order to estimate the behaviour of the system under different operational conditions. In this paper, two models were developed based on ANNs to predict the TOC removal percentage and phenol degradation percentage. The proposed models consist three layers with ten neurons in the hidden layer, and were optimized to predict TOC and phenol removal percentage with highest accuracy. The models provided good estimates for the TOC and phenol degradation, and showed that neural network modelling could successfully reproduce experimental data and predict the behaviour of the process.
3. The use of ANN as statistical tool permitted to predict the Fentonic removal of TOC and phenol. All of the studied parameters in this work (initial concentration of the phenol and H_2_O_2_, initial catalyst and reaction time) have considerable effects on the degradation efficiency and, as expected, the initial concentration of H_2_O_2_ with a relative importance of 23–24%, appeared to be the most influential parameter in the degradation process.

The ANN modeling practice has several favorable features such as efficiency, generalization and simplicity, which makes it an attractive choice for modeling complex systems, such as wastewater treatment processes; which also has the potential to be used as an on-line automatic control approach. This information is essential for the adequate scale-up and design of industrial scale batch reactors for the treatment of organic contaminants in wastewaters.
